# Sensitivity of genome-wide tests for mitonuclear genetic incompatibilities

**DOI:** 10.1101/2025.06.30.662443

**Published:** 2025-07-04

**Authors:** Shady A. Kuster, Molly Schumer, Justin C. Havird, Daniel B. Sloan

**Affiliations:** 1 Cell and Molecular Biology, Colorado State University; 2 Department of Biology, Colorado State University; 3 Department of Biology, Stanford University; 4 Freeman Hrabowski Fellow, Howard Hughes Medical Institute; 5 Department of Integrative Biology, University of Texas at Austin

## Abstract

Mismatches between interacting mitochondrial and nuclear gene products in hybrids have been proposed to disproportionately contribute to the formation of early species boundaries. Under this model, genetic incompatibilities emerge when mitochondrial haplotypes are placed into a cellular context without their coevolved nuclear-encoded mitochondrial (n-mt) proteins. Although there is strong evidence that mitonuclear coevolution has contributed to reproductive isolation in some cases, it is less clear how far-reaching the effects of mitonuclear incompatibilities are in speciation. Does disrupting co-adapted mitonuclear genotypes have broad, genome-wide effects with numerous n-mt loci contributing to reproductive isolation? We leverage a system with several hybridizing species pairs (*Xiphophorus* fishes) that have known mitonuclear incompatibilities of large effect to ask whether a general signal of incompatibility is present when considering all n-mt genes. After dividing nuclear-encoded proteins into three classes based on level of interaction with mitochondrial gene products, we found only inconsistent statistical evidence for a difference between these classes in the degree of conserved mitonuclear ancestry. Our results imply that genome-wide scans focused on enrichment of broad functional gene classes may sometimes be insufficient for detecting a history of mitonuclear coevolution, even when strong selection is acting on mitonuclear incompatibilities at multiple loci.

## INTRODUCTION

Since its endosymbiotic origin, the mitochondrion has evolved into an integral component of the eukaryotic cell, functioning in such processes as oxidative phosphorylation (OXPHOS), Ca^2+^ acquisition and signaling, and biosynthesis of heme and iron-sulfur clusters. Bilaterian metazoan mitochondrial genomes (mitogenomes) have been reduced to encode only 13 core protein subunits of the electron transport chain, 2 rRNAs, and 22 tRNAs, making mitochondria dependent on the import of nuclear-encoded, but mitochondrially targeted, (n-mt) proteins for all functions [1–4].

Mitogenomes evolve at a faster rate than nuclear genomes in most bilaterian metazoan systems [5–8]. These elevated substitution rates may result from a higher underlying mutation rate in combination with inefficient purging of deleterious mutations due to smaller effective population size, lack of recombination, and uniparental inheritance [9–12], although explanations based on reduced efficacy of selection in mitogenomes have been recently challenged [13–15]. Regardless of the cause, the consequences of high mitochondrial DNA (mtDNA) substitution rates are often deleterious [16]. As such, substitutions in mitochondrially encoded proteins may create selection for compensatory changes in interacting n-mt proteins [17–19], and signatures of coevolution have indeed been found between the mitochondrial and nuclear genomes [20–23]. Other forms of mitonuclear coevolution (i.e., reciprocal selection for compatible genotypes) are also possible [20,24–26]. Regardless of the exact process, the outcome is a set of interacting proteins that are functionally dependent on sufficient “matching” of lineage-specific amino acid substitutions in mitochondrial and n-mt proteins [24].

As a result, disrupting the association between a mitochondrial haplotype and its coevolved nuclear counterparts (e.g., via secondary contact following speciation) is predicted to uncover genetic incompatibilities that made early contributions to reproductive isolation [5,27]. Genes that evolve faster are theorized to be the first in generating incompatibilities between diverging populations [28]. Given the rate at which mitonuclear genes coevolve and importance of mitochondrial function to cellular metabolism, mitonuclear incompatibilities are proposed to have a disproportionate effect on the accumulation of reproductive barriers during speciation [24,29,30]. Although studies have found evidence of mitonuclear incompatibilities (e.g., [31,32]) in some systems, it is still unclear how far-reaching these effects are. In other words, when selection is acting to maintain “matched” mitonuclear genotypes, are relatively few nuclear loci of large effect involved or many loci of small effect? Moreover, do n-mt genes preferentially contribute to mitonuclear genetic incompatibilities? We hypothesize that, relative to other nuclear genes, n-mt genes are more strongly affected by selection acting to maintain associations between matched lineage-specific mitonuclear genotypes. Therefore, examining signatures of mtDNA “match” in n-mt genes vs. nuclear-encoded genes that lack mitochondrial interactions (non-n-mt genes) should reveal a history of mitonuclear coevolution across the genome at species boundaries.

Species of the swordtail fish genus *Xiphophorus* have recently begun hybridizing in multiple river systems in Mexico, providing a model of repeated hybridization and speciation dynamics [33–42]. Recent work on hybrid zones between sister species *X. birchmanni* and *X. malinche* has identified severe mitonuclear incompatibilities involving the *ndufs5* and *ndufa13* n-mt genes [43]. Mitonuclear mismatches at these loci have severe fitness effects, including death during embryonic development. Subsequent investigations have identified that at least nine nuclear genomic regions (seven of which contain a total of 10 n-mt genes) are involved in mitonuclear incompatibilities in these hybrids [44]. Some of these incompatibilities, including the *ndufs5* and *ndufa13* n-mt genes, were also found in *X. birchmanni* and *X. cortezi* hybrids [42]. We therefore leveraged this system to investigate evidence for mitonuclear matching on a genome-wide scale in hybridization and the ability to detect these interactions with commonly used approaches to partition nuclear genes into classes based on their role in mitochondrial function. We categorized nuclear genes into three groups based on whether the encoded protein (1) interacts with mitogenomic gene products (interacting n-mt), (2) functions in mitochondria but does not physically interact with mitogenomic gene products (non-interacting n-mt), or (3) does not function in mitochondria (non-n-mt). This approach allows for an unbiased test of whether selection for matched mitonuclear genotypes leaves genome-wide signatures based on this *a priori* partitioning of genes into mitochondrial functional categories. We found inconsistent statistical evidence for differences among these categories for metrics of selection on mitonuclear interactions in *Xiphophorus* hybrids, indicating that the role of n-mt genes (either interacting or non-interacting) is not always detectable on a genome-wide scale.

## METHODS

### Genomic data from *Xiphophorus* hybrid populations

Three datasets resulting from previous local ancestry inference in hybrid *Xiphophorus* fishes were acquired from [37,40,43]. These datasets were originally analyzed using the *ancestryinfer* pipeline and resulted in ancestry probabilities at between 628,881 and 689,982 sites genome-wide (Table 1). Each dataset includes samples from a population of hybrid *Xiphophorus* individuals inhabiting sections of rivers in Hidalgo Mexico or San Luis Potosí Mexico. The first (Calnali Low, or CALL), is on the Calnali River and consists of *X. birchmanni* and *X. malinche* hybrids. The second is at Chahuaco Falls (CHAF) on the Calnali River. It is separated from the CALL population by a waterfall (~50m tall) [43] and consists of hybrids of the same species as CALL. The third consists of two populations (Huextetitla and Santa Cruz, or HUEX-STAC) downstream of the same fork on the Santa Cruz River and derived from a single initial hybridization event between *X. birchmanni* and *X. cortezi* followed by independent evolution [37,38].

Ancestry informative markers (AIMs) across the nuclear and mitochondrial genomes were assigned a posterior probability of ancestry to each parental species, as described previously [36,39,45]. Informative sites were defined as those with at least a 98% frequency difference between the parental species, as previously described [33,44]. We used a published annotation of the *X. birchmanni* reference genome [46] to identify genes containing each AIM. To obtain average nuclear ancestry and mitochondrial haplotype frequency for each population, we first filtered posterior probabilities of AIMs so they were either below 0.2 or above 0.8 to ensure high quality calls. These filtered probabilities were then averaged for nuclear and mitochondrial AIMs, respectively (Table 1).

### Identification of *Xiphophorus* n-mt genes

The gffread [47] program was used to translate the *X. birchmanni* fasta file into protein sequences, using the -V option to remove any coding sequence with an in-frame stop codon. To annotate n-mt proteins, we compared the *Xiphophorus* translated proteome to UniProt human (UP000005640, GCA_000001405.27) and mouse (UP000000589, GCA_000001635.8) protein sequences. N-mt proteins have been empirically identified in humans and mice as part of the MitoCarta 3.0 project [4]. Because the UniProt human and mouse datasets differed in nomenclature from the MitoCarta dataset, we assigned protein names by searching for MitoCarta sequences in the UniProt human sequences with BLASTP v2.15.0+. A custom Perl script was then used to parse the BLASTP output, and identified proteins were manually confirmed to be the correct protein if percent coverage and sequence identity were not 100%. OrthoFinder v2.5.4 [48] was then run with the primary transcript for each *Xiphophorus* sequence and the full sets of human and mouse protein sequences.

The resulting orthogroups were then organized into our three gene classes: (1) interacting n-mts, (2) non-interacting n-mts, and (3) non-n-mts. MitoCarta proteins differentiated non-n-mt from n-mt proteins. Interacting and non-interacting proteins were distinguished based on a previously published [49] list of interacting n-mt proteins that includes OXPHOS subunits, ribosomal subunits, aminoacyl-tRNA synthetases, and DNA and RNA polymerases, with updates to match the most recent empirical evidence [4] (Supplemental Table 1. List of human interacting n-mt genes used in this study, modified from Supplemental Table 1). Comparison of OrthoFinder output to these two gene lists identified *X. birchmanni* proteins that have human and mouse orthologs.

### Linkage disequilibrium calculation

We used mitonuclear linkage disequilibrium (LD) as a metric for the association between mitochondrial and nuclear alleles in hybrid populations. However, the mitogenome is fixed for the haplotype from just one of the parental species (*X. cortezi*) in HUEX-STAC [38], so mitonuclear LD could only be calculated for the CALL and CHAF populations (see below for HUEX-STAC analysis methods). Calculations were performed using the $D^{\prime }$ and $r^{2}$ statistics [50]. In the equations that follow, A represents an n-mt locus and B the mitogenome, and pA and pB represent their respective allele frequencies. pAB is the frequency of the haplotype AB across the two sites. The A and B alleles were assigned to be from the same parental population so that positive and negative values of LD indicate a higher frequency of parental or recombinant allele combinations, respectively.

$$D=p_{AB}-p_{A}p_{B}$$

$$D^{\prime }=\frac{D}{D_{max}}$$

where

$$D_{max}=\left\{\begin{array}{l} min\left[p_{A}\left(1-p_{B}\right),\left(1-p_{A}\right)p_{B}\right]\;\mathrm{if}\;D>0\\ min\left[p_{A}p_{B},\left(1-p_{A}\right)\left(1-p_{B}\right)\right]\;\mathrm{if}\;D<0 \end{array}\right.$$

and

$$r^{2}=\frac{D^{2}}{p_{A}\;*\;\left(1\;-\;p_{A}\right)\;*\;p_{B}\;*\;\left(1\;-\;p_{B}\right)}$$

Because all mitochondrial genes are linked on the same non-recombining mitogenome, a single AIM was used (CALL and CHAF’s AIM was at position 45 of the reference genome, GenBank accession CM071424.1, in *COX2*, and HUEX-STAC’s AIM was at position 846 in *ATP6*) to identify the mitochondrial haplotype for each fish and to represent the mitogenome in all LD analyses. This approach was appropriate for our data, as all mtDNA AIMs had approximately the same posterior probability for ancestry assignment. Posterior probabilities were summed across each nuclear AIM as a proxy for frequency of the two alleles. The two LD statistics were calculated for each protein class separately in a custom R script. LD values were visualized using ggplot2 v3.4.0 and ggridges v0.5.4 in RStudio v2023.03.1+446.

LD values were then averaged across AIMs within each nuclear gene to limit pseudo-replication. A one-way ANOVA from the R stats v4.2.2 package (commands *lm* and *anova*) was used to determine whether protein class had a statistically significant impact on LD values. Summary statistics were collected using commands *n*, *mean*, and *sd* from the dplyr v1.1.0, base v4.2.2, and stats v4.2.2 packages, respectively. We also identified the AIMs and genes with the top 1% of LD values with a custom R script.

N-mt genes known to be involved in mitonuclear incompatibilities were identified from [44]. We tracked the location of these genes in the distribution of $D^{\prime },\;r^{2}$, and allele frequency values for the respective populations. To determine how these genes compared to the mean value, their statistic was standardized to the mean by a Z-score calculation.

### HUEX-STAC allele frequency calculation

The HUEX-STAC populations are both fixed for the *X. cortezi* mitochondrial haplotype, so to see how mtDNA background affects selection on n-mt genes in this system, ancestry frequency was calculated at interacting n-mt, non-interacting n-mt, and non-n-mt loci. These values were averaged within each gene. Allele frequencies were identified and visualized using a custom R script. A one-way ANOVA and summary statistics were computed following the same process as described in the previous section.

A permutation test was then performed to determine whether significant results were robust to violations of normality assumptions in ANOVA. Allele frequency values were permutated by randomly reassigning them across genes 9999 times using base R v4.2.2 functions in a custom R script. Permuted datasets were then used to calculate null expectations for differences in allele frequencies between interacting n-mt and non-n-mt gene classes. The observed difference was compared to the resulting distribution to determine the probability of our result having arisen by chance.

### Data and code availability

All code used in this project has been deposited in GitHub at https://github.com/sakuster/sensitivity-of-genome-wide-mtnuc. Data can be accessed on figshare (https://doi.org/10.6084/m9.figshare.c.7902011.v1).

## RESULTS

### Identification of *Xiphophorus* n-mt sequences

Of the 24,269 *Xiphophorus* annotated genes, 86.5% (20,988) were assigned to an orthogroup by OrthoFinder. We found that 75.5% (13,264) of the 17,566 identified orthogroups contained at least one *Xiphophorus* gene, and 69.4% (12,198) included at least one gene from all three species (*X. birchmanni*, mouse, and human). Gene duplications during the divergence of these three species resulted in some orthogroups with multiple *X. birchmanni* genes (24.1% of orthogroups, or 4,234, contained 2 or more).

These orthogroups were used to classify *Xiphophorus* genes into our three functional categories for each population (Table 2). MitoCarta 3.0 consists of 1,136 human and 1,140 mouse n-mt genes. All but three of the mouse n-mt genes (*Gm4984*, *Htd2*, and *Nsun4*) were identified, and only one human n-mt gene (*NDUFB1*) was not identified in *X. birchmanni*. Overall, 159 orthogroups were found to contain interacting n-mts, 822 to contain non-interacting n-mts, and 15,545 to contain non-n-mts. These orthogroups had 184, 1,042, and 23,043 *X. birchmanni* genes, respectively. We then determined which of these genes overlapped with the AIMs in our three hybrid datasets (Table 2).

### Mitonuclear linkage disequilibrium is high but does not differ among gene classes in hybrid populations with segregating mitochondrial haplotypes

**Figure 1.**
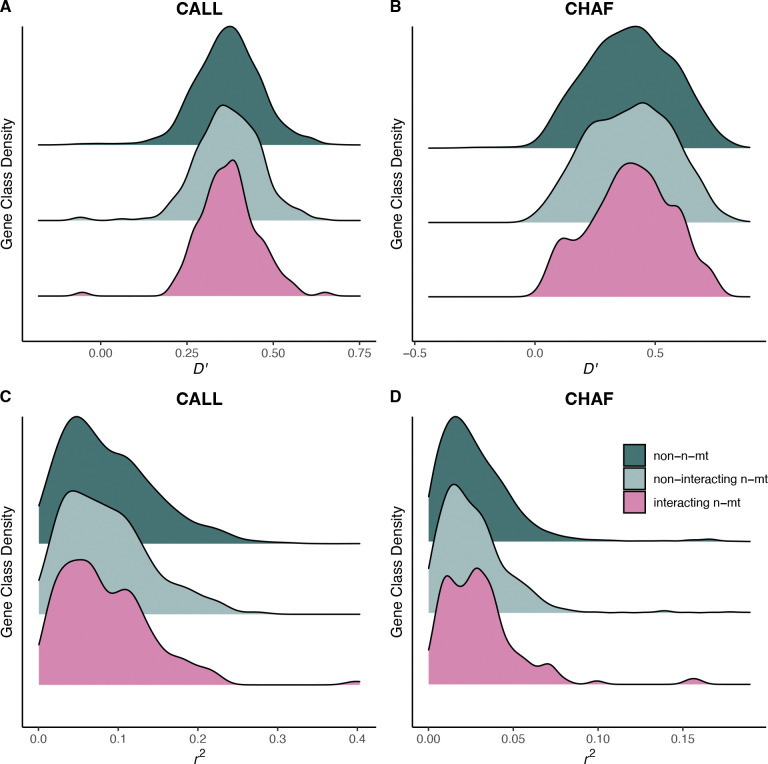
*Distribution of mitonuclear LD values for each gene class in the CALL (A and B) and CHAF (C and D) populations. LD is calculated as $D^{\prime }$ in panels A and C and as* r^2^ *in panels B and D. Note the change of scale on the x-axis across populations and LD statistics. P-value for a one-way ANOVA testing if gene class affects LD value: CALL $D^{\prime }\;0.94\;(A),\;CHAF\;D^{\prime }$ 0.99 (B), CALL* r^2^ *0.45 (C), CHAF* r^2^ *0.34 (D). Summary statistics are reported in* Supplemental Table 2.

**Figure 2.**
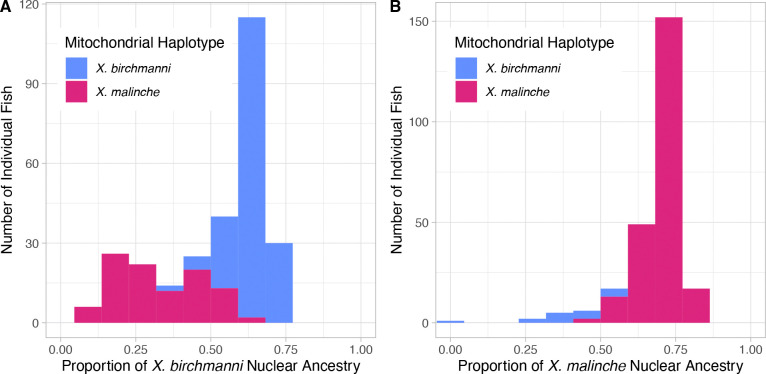
*Stacked bar histogram for the proportion of the nuclear genome derived from the major parent for each fish in A) CALL and B) CHAF. Proportion is calculated as the sum of all major parent AIMs divided by number of AIMs. Each fish is colored by which of the two segregating mitochondrial haplotypes it has (blue =* X. birchmanni, *fuchsia =* X. malinche*). Note that y-axis upper limits differ between the panels.*

We sought to determine whether n-mt genes maintained higher mitonuclear LD in *Xiphophorus* hybrids due to selection against mitonuclear incompatibilities. On average, mitonuclear LD values were high across the entire nuclear genome in the CALL and CHAF hybrid populations (Figure 1), with nuclear ancestry matching the mitochondrial haplotype (positive $D^{\prime }$ values). This result is also illustrated by differences in average nuclear ancestry between individuals with mitochondrial haplotypes from the two possible parent species (Figure 2). These strong associations are likely due to population structure in these two populations [33,42].

Under a model of mitonuclear coevolution, we predicted that the interacting n-mt protein class would have higher mitonuclear LD values compared to the other protein classes. However, we did not find a significant difference between the protein classes for CALL ($D^{\prime }$: *p* = 0.936; $r^{2}$: *p* = 0.446) or CHAF ($D^{\prime }$: *p* = 0.994; $r^{2}$: *p* = 0.341; summary statistics in Supplemental Table 2). Therefore, it does not appear that mitochondrial haplotypes have preferentially maintained associations with n-mt loci at a genome-wide scale in these hybridizing populations.

One possible explanation for the lack of observed differences in mitonuclear LD among gene classes in the CALL and CHAF populations is that selection on a small number of n-mt genes may not lead to detectable shifts in genome-wide averages but could still result in individual loci with the strongest association with the mitogenome being enriched for interacting n-mt genes. To test this possibility, we took the top 1% of all genes based on our association metrics ($D^{\prime }$ and $r^{2}$) and tested for overrepresented gene classes. However, none of these gene sets showed significant enrichment for interacting n-mt genes (Supplemental Table 4).

Previous analyses of mitonuclear incompatibilities in *X. birchmanni* x *X. malinche* hybrids in the CALL population described nine incompatible nuclear genomic regions containing 10 n-mt genes [43,44]. Eight of these 10 genes were identified in our CALL analysis, and five (*MTERF4*, *NDUFA13*, *NDUFS5*, *RMDN3*, *SMIM8*) of those eight genes were outliers for mitonuclear LD (with a Z-score of >2 above the mean $D^{\prime }$ value) (Supplemental Table 3). Therefore, although the top 1% genome-wide LD values did not show an overrepresentation of n-mt genes, we do find that some genes of known biological importance exhibit signatures of elevated mitonuclear LD. Two other genes (*LYRM2* and *MMUT*) were also above the mean but not outliers by this definition, and the final gene (*UQCRC2*) was very close to the mean. Repeating these analyses with $r^{2}$ values instead of $D^{\prime }$ values resulted in slight changes, but the results were generally similar (Supplemental Table 3). Overall, our results show that n-mt genes previously found to be involved in mitonuclear incompatibilities show the expected trend towards higher mitonuclear LD values, but using $D^{\prime }$ or $r^{2}$ alone would have been insufficient to identify these genes as the most significant outliers in the genome (Supplemental Figure 1, Supplemental Figure 2).

To see whether these genetic incompatibilities showed similar signatures across populations with varying mtDNA frequencies and different parental source species, we also investigated mitonuclear associations for CHAF and HUEX-STAC (Supplemental Table 3, Supplemental Figure 3, Supplemental Figure 4, Supplemental Figure 5). The CALL and CHAF populations, which derive from the same parental species, exhibit a significant correlation in mitonuclear LD ($D^{\prime }$) across this set of incompatibility genes (Pearson correlation; *r* = 0.89; *p* = 0.0012). Comparisons with allele frequencies in HUEX-STAC, which originates from a different pairing of parental species, also show a positive trend, but the relationships are weaker and non-significant for both CALL (*r* = 0.35; *p* = 0.39) and CHAF (*r* = 0.68; *p* = 0.06). Interestingly, the two genes that exhibit an incompatibility with *X. malinche* mtDNA (*MTERF4, NDUFA13*) are found to have some of the strongest LD signatures in CHAF (Supplemental Table 3), which has a high frequency of *X. malinche* mtDNA (Table 1).

### Interacting n-mt genes show enriched mitochondrial match in an older hybrid population fixed for a mitochondrial haplotype

**Figure 3.**
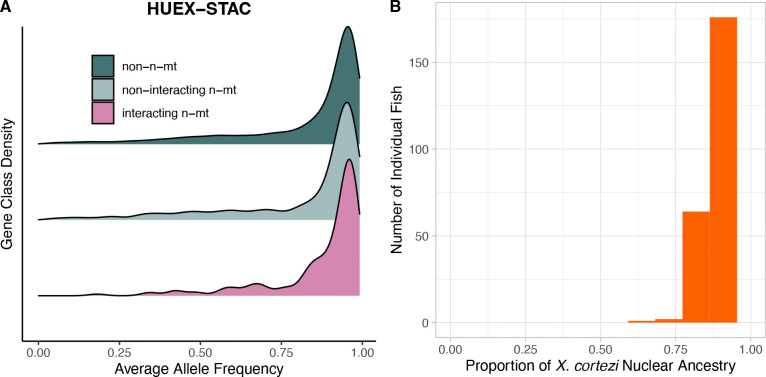
*HUEX-STAC analyses for A) allele frequencies for each gene class and B) nuclear genome-wide ancestry for the HUEX-STAC population, a hybrid population fixed for the* X. cortezi *mitogenome.*

The HUEX-STAC populations are fixed for the *X. cortezi* mitochondrial haplotype, so we expected to see *X. cortezi* ancestry disproportionately represented at n-mt loci. HUEX-STAC individuals generally have a large percentage of *X. cortezi* ancestry across their entire nuclear genome with a mean allele frequency of 82.7% ± a standard error of 0.001% (Figure 3). Non-interacting n-mt and non-n-mt genes showed similar mean *X. cortezi* allele frequencies at 81.8 ± 0.07% and 82.7 ± 0.01%, respectively. Interacting n-mt genes had a higher mean allele frequency (87.4 ± 0.01%, Figure 3). Thus, unlike in the CALL and CHAF populations, individuals collected from the HUEX-STAC population did demonstrate a difference in allele frequencies among gene classes (one way ANOVA: *p* = 0.005) and supported the predicted higher degree of matched mitonuclear ancestries for interacting n-mt genes (Figure 3). This result was also supported by a non-parametric permutation test (Supplemental Figure 6).

## DISCUSSION

Because loci that have faster rates of evolution are expected to cause genetic incompatibilities between diverging populations, mtDNA, which evolves on average 19× faster than nuclear DNA in vertebrates [8], is proposed to play an important role in the evolution of incompatibilities [30]. Higher rates of genome evolution in mtDNA can result in corresponding compensatory mutations in n-mt genes [17,19–21,51]. Some investigators have taken to splitting up the nuclear genome into n-mt and non-n-mt portions to investigate the effects of mitonuclear interactions and test for a disproportionate role of n-mt genes (Supplemental Table 5). However, it is unclear how sensitive this approach is, so we applied it to a system known to have severe effects of mitonuclear incompatibilities involving multiple loci.

Here, we specifically tested the prediction that the interacting n-mt gene class would show the strongest “matched” association with mitochondrial haplotype. Despite the known role of n-mt genes in hybrid incompatibilities between *X. birchmanni* and *X. malinche*, the CALL and CHAF populations did not support this prediction. This negative result likely speaks to the statistical power issues inherent in identifying mitonuclear interactions. The sensitivity of genome-wide tests for signatures of selection acting preferentially on n-mt genes will likely be affected by the number of genes under selection and/or where the n-mt genes are distributed in the genome – factors that are also important for detecting loci involved in nuclear-nuclear hybrid incompatibilities [52].

N-mt genes make up a small portion of the genome, and only a subset of those genes may be participating in mitonuclear epistasis. Incompatibilities can potentially involve a few loci with a large effect size or many loci of small effect. The analyses performed by Robles et al. [44] identified 10 n-mt genes among nine genomic regions contributing to mitonuclear incompatibilities in *Xiphophorus* species hybrid populations, one of which was CALL. These genes represent only 6.8% of all interacting n-mt genes and 1.0% of all n-mt genes. Selection on these few genes would have to be exceptionally strong and have had enough time to act to detect a significant shift in the average for the entire interacting n-mt gene class. Therefore, even in cases where n-mt genes are contributing to negative mitonuclear epistasis, the number of loci may be too small to detect genome-wide effects with comparisons such as those implemented here, which are inherently designed to detect polygenic contributions to incompatibilities. In contrast, systems such as *Tigriopus* copepods, in which many genes are involved in mitonuclear incompatibilities, have proven to be very powerful in advancing the field of mitonuclear genetics and genomics [21,31].

Background nuclear-nuclear LD and the distribution of n-mt genes across the genome will also affect the ability to detect a difference in mtDNA ancestry matching between n-mt and non-n-mt genes. Any signal of this difference arising from selection on an individual n-mt target could be diluted by hitchhiking at linked non-n-mt loci [53]. This effect would be particularly problematic for populations that are the result of recent admixture events, such as CALL and CHAF (initial admixture ~46 generations ago [35]), and therefore, have not had many generations for recombination to break up haplotype blocks in the nuclear genome [41,54]. In some cases, clustering of n-mt genes in specific regions of the genome could create strong targets of selection that overcome this dilution effect. For example, genomic regions with a high density of n-mt genes have been identified in the long-tailed finch [55] and the eastern yellow robin [56], both of which have found evidence that n-mt genes disproportionately share ancestry with mtDNA.

Additionally, not all mitonuclear incompatibilities are necessarily mediated by n-mt genes. Instead, some incompatibilities may involve nuclear-encoded proteins that are not targeted to mitochondria but nonetheless create genetic interactions with mtDNA through co-functionality in biochemical and signaling pathways that span multiple cellular compartments [51]. Any contributions of non-n-mt genes to mitonuclear incompatibilities would directly undermine search strategies based on partitioning the genome into n-mt and non-n-mt categories.

Despite these challenges, one of the three hybrid populations we investigated (HUEX-STAC) supported our prediction of enrichment in matched ancestry for n-mt genes. Notably, this hybrid population is ~217 generations older than CALL and CHAF [33,35], and it is the only one of the populations we investigated that is fixed for a mitochondrial haplotype from just one of the parental species. Its older age means there has been more time for incompatible genotype combinations to be purged from the population and for recombination to have broken up haplotype blocks in the nuclear genome, thereby limiting the effects of hitchhiking. We therefore speculate that the age of HUEX-STAC and the fixation of a mitochondrial haplotype may have made selection on n-mt loci stronger and/or more effective. In such populations, half of the possible mitonuclear combinations have been removed, so selection for matched ancestry would consistently favor the same n-mt allele. In contrast, when mitochondrial haplotypes are still segregating, the fitness effects of an n-mt allele may change depending on an individual’s mtDNA. Because mitochondrial and nuclear loci are not physically linked, very strong fitness effects would be needed to preserve mitonuclear LD in segregating populations [57].

Although there is an enormous body of literature investigating mitonuclear interactions, the number of studies that specifically test for preferential effects on associations involving n-mt genes in the context of hybridization is much more limited. We identified 16 previous studies that specifically tested this prediction (Supplemental Table 5). In these 16 studies, 23 different hybrid populations or species with an ancient history of admixture were used, and nine of those 23 found support for preferential effects on n-mt genes. However, we found examples that both support and oppose the hypothesis that fixation of a mitochondrial haplotype strengthens selection on n-mt genes. Expanding the availability of such genomic datasets from hybrid systems and performing a formal meta-analysis would be important future directions to assess relevant variables, such as whether mitochondrial haplotypes are segregating in the population, the age of the hybrid population, the number of loci involved and their effect sizes, and the genetic structure of the population. Reanalysis of publicly available genome-level data from hybrid populations may also be a valuable approach to control for method of ancestry inference and metric of association between mtDNA and nuclear loci.

Another variable that differentiates the HUEX-STAC population is the parental makeup of the hybrids. Although geographically distinct, CHAF and CALL share parental species (*X. birchmanni* and *X. malinche*). In contrast, HUEX-STAC was formed by hybridization between *X. birchmanni* and *X. cortezi*. These three species demonstrate mitonuclear discordance, as *X. malinche* and *X. cortezi* mitogenomes are more closely related to each other, but the *X. malinche* nuclear genome is sister to *X. birchmanni* [58]. Therefore, hybrids in the HUEX-STAC population have nuclear genomes that are more divergent, on average, from one another than in the CALL and CHAF populations. Furthermore, recent work has shown that the *X. cortezi*, *X. malinche*, and *X. birchmanni* species have a complex evolutionary history that involves the introgression of n-mt genes *ndufs5* and *ndufa13* from *X. malinche* into *X. cortezi*, which produces similar incompatibility phenotypes in hybrids between *X. cortezi* and *X. birchmanni* [42]. HUEX-STAC hybrids are then faced with nuclear alleles that are farther diverged from one another in combination with a prior history of introgression contributing to mitonuclear incompatibilities. These two factors may cause stronger and more widespread incompatibilities involving n-mt genes, resulting in stronger selection for matched mitonuclear ancestry. Given these different demographic histories, each of the hybrid populations we analyze here is expected to have numerous unique features, so caution is warranted in attributing any of our results to a single feature (such as the fixed mitochondrial haplotype in HUEX-STAC). Nevertheless, we view the potential role of fixed vs. segregating mitochondrial haplotypes to be an important area of future research given the small number of studies conducted to date.

Overall, our findings suggest that splitting up the genome into n-mt and non-n-mt gene classes may be an underpowered method to detect effects of mitonuclear epistasis in recently admixed populations, even when there are clear biological effects of said epistasis. Although such tests for genome-wide signatures may offer a relatively unbiased approach, they may be susceptible to false negatives. Therefore, important contributions of mitonuclear interactions to speciation may remain unrecognized in many systems.

## Supplementary Material

Supplement 1

## Figures and Tables

**Table 1.** Summary of Xiphophorus hybrid populations used in this study.

Site Abbreviation	CALL (Calnali Low)	CHAF (Chahuaco Falls)	HUEX-STAC (Huextetitla and Santa Cruz)
**Population Site**	Calnali River	Calnali River	Santa Cruz River
**Major Parent Species**	*X. birchmanni*	*X. malinche*	*X. cortezi*
**Minor Parent Species**	*X. malinche*	*X. birchmanni*	*X. birchmanni*
**Number of Individuals**	281	250	255
**Mitochondrial Haplotype Frequency** ^a^	63.7% *X. birchmanni*	93.6% *X. malinche*	99.6% *X. cortezi*
**Average Major Parent Nuclear Ancestry**	52.2%	68.2%	84.6%
**Nuclear Ancestry Informative Sites**	629,430	628,788	689,966
**Mitochondrial Ancestry Informative Sites**	93	93	16
**Estimated Generations Since Initial Admixture**	46 ± 1 [40]	46 ± 1 [40]	263 [33]

a Haplotype frequencies for each population were calculated following filtering of mitochondrial AIMs with low-confidence genotypes (posterior probability between 0.2 and 0.8). Filtering resulted in sample sizes slightly lower than the Number of Individuals row: CALL n = 278, CHAF n = 249, HUEX-STAC n = 244.

**Table 2.** *AIMs and genes belonging to each protein class. The orthogroup row indicates how many orthogroups containing* X. birchmanni *protein sequences were found for each gene class.*

Population	Interacting n-mt	Non-interacting n-mt	Non-n-mt
**Orthogroups**	159	822	15,545
**CALL AIMs (unique genes)**	954 (148)	8,580 (883)	287,834 (19,312)
**CHAF AIMs (unique genes)**	952 (148)	8,575 (883)	287,592 (19,307)
**HUEX-STAC AIMs (unique genes)**	997 (162)	9,399 (947)	319,679 (20,544)
