# Supplementary material for: Sensitivity of genome-wide tests for mitonuclear genetic incompatibilities
Source: bioRxiv. 2025 Jul 4:2025.06.30.662443. Preprint. [Version 1] doi: 10.1101/2025.06.30.662443 (PMC12236695; doi:10.1101/2025.06.30.662443)
Supplement: Supplement 1 [file NIHPP2025.06.30.662443v1-supplement-1.pdf]

bioRxiv preprint doi: https://doi.org/10.1101/2025.06.30.662443; this version posted July 4, 2025. The copyright holder for this preprint (which was not certified by peer review) is the author/funder, who has granted bioRxiv a license to display the preprint in perpetuity. It is made available under aCC-BY 4.0 International license.

SUPPLEMENTAL FIGURES

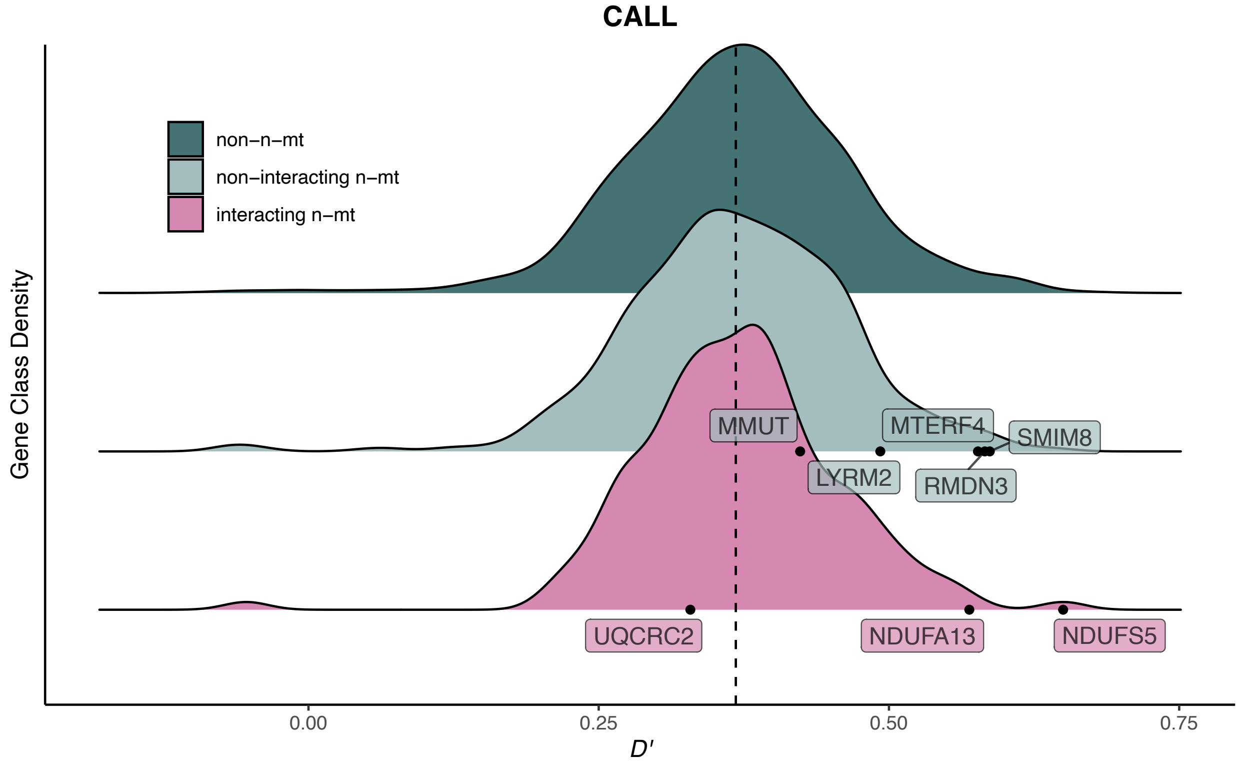

Supplemental Figure 1. Location of previously identified incompatibility genes [44] in the CALL population D' *values. Dashed line indicates genome-wide mean* D'.

bioRxiv preprint doi: https://doi.org/10.1101/2025.06.30.662443; this version posted July 4, 2025. The copyright holder for this preprint (which was not certified by peer review) is the author/funder, who has granted bioRxiv a license to display the preprint in perpetuity. It is made available under aCC-BY 4.0 International license.

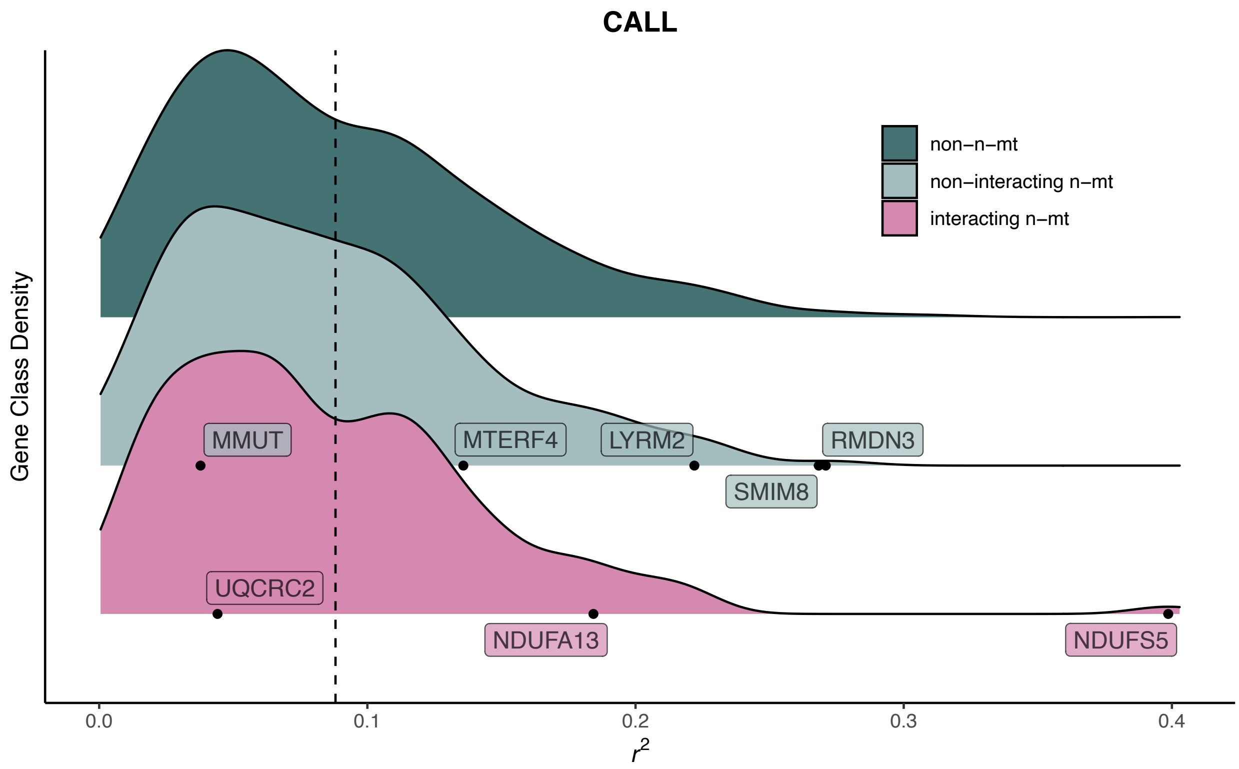

Supplemental Figure 2. Location of previously identified incompatibility genes [44] in the CALL population r^2 *values. Dashed line indicates genome-wide mean* r^2.

bioRxiv preprint doi: https://doi.org/10.1101/2025.06.30.662443; this version posted July 4, 2025. The copyright holder for this preprint (which was not certified by peer review) is the author/funder, who has granted bioRxiv a license to display the preprint in perpetuity. It is made available under aCC-BY 4.0 International license.

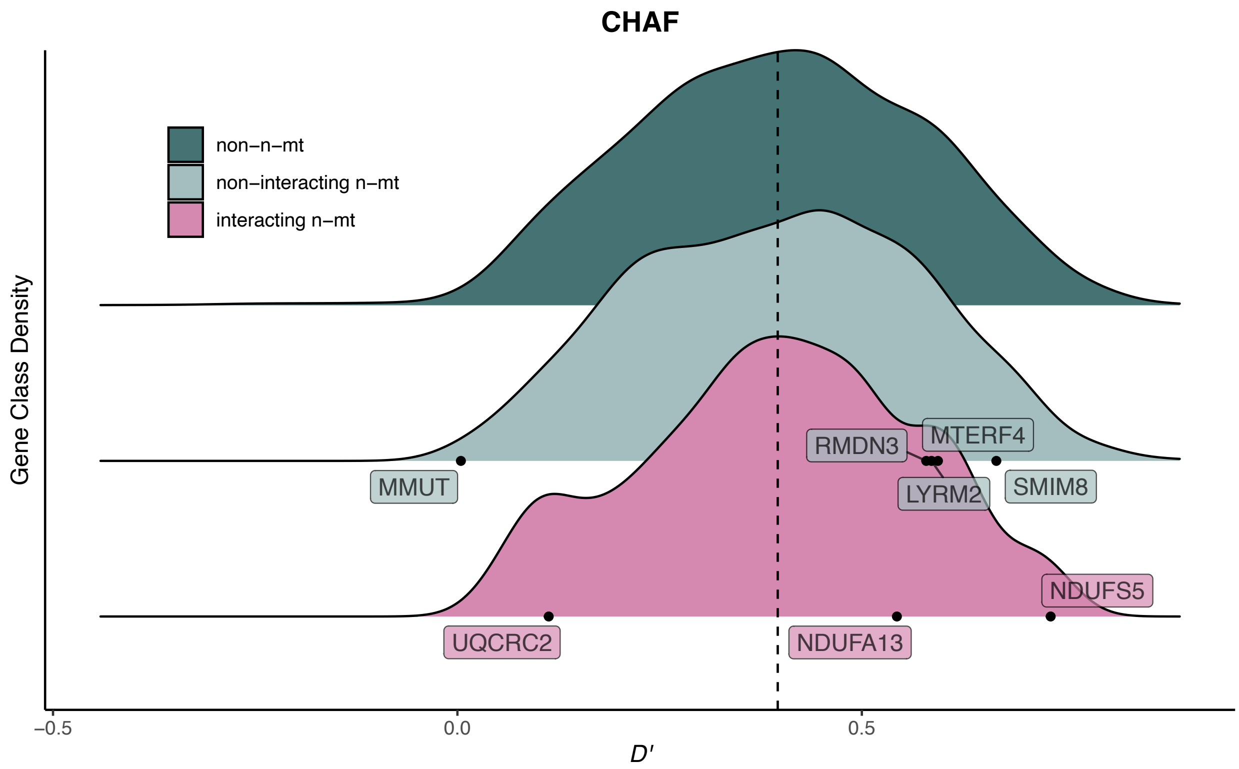

Supplemental Figure 3. Location of previously identified incompatibility genes [44] in the CHAF population D' *values. Dashed line indicates genome-wide mean* D'.

bioRxiv preprint doi: https://doi.org/10.1101/2025.06.30.662443; this version posted July 4, 2025. The copyright holder for this preprint (which was not certified by peer review) is the author/funder, who has granted bioRxiv a license to display the preprint in perpetuity. It is made available under aCC-BY 4.0 International license.

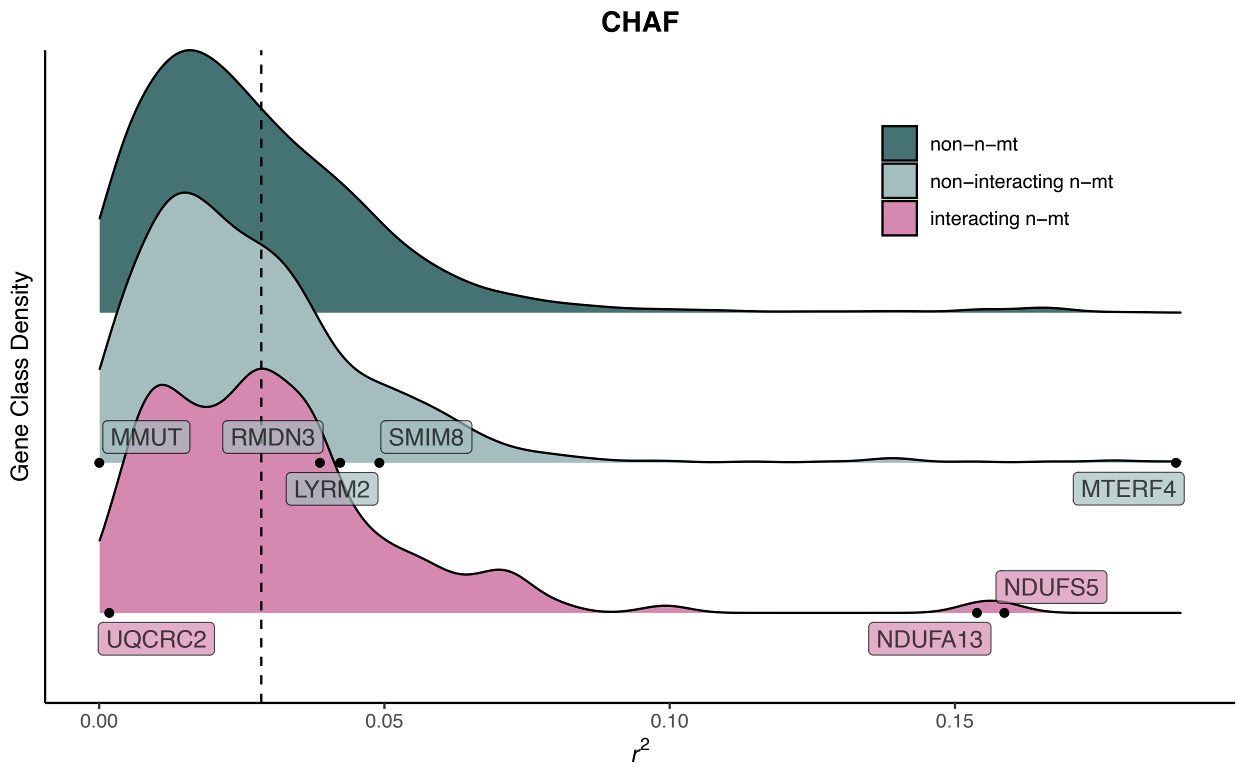

Supplemental Figure 4. Location of previously identified incompatibility genes [44]in the CHAF population r^2*values. Dashed line indicates genome-wide mean* r^2.

bioRxiv preprint doi: https://doi.org/10.1101/2025.06.30.662443; this version posted July 4, 2025. The copyright holder for this preprint (which was not certified by peer review) is the author/funder, who has granted bioRxiv a license to display the preprint in perpetuity. It is made available under aCC-BY 4.0 International license.

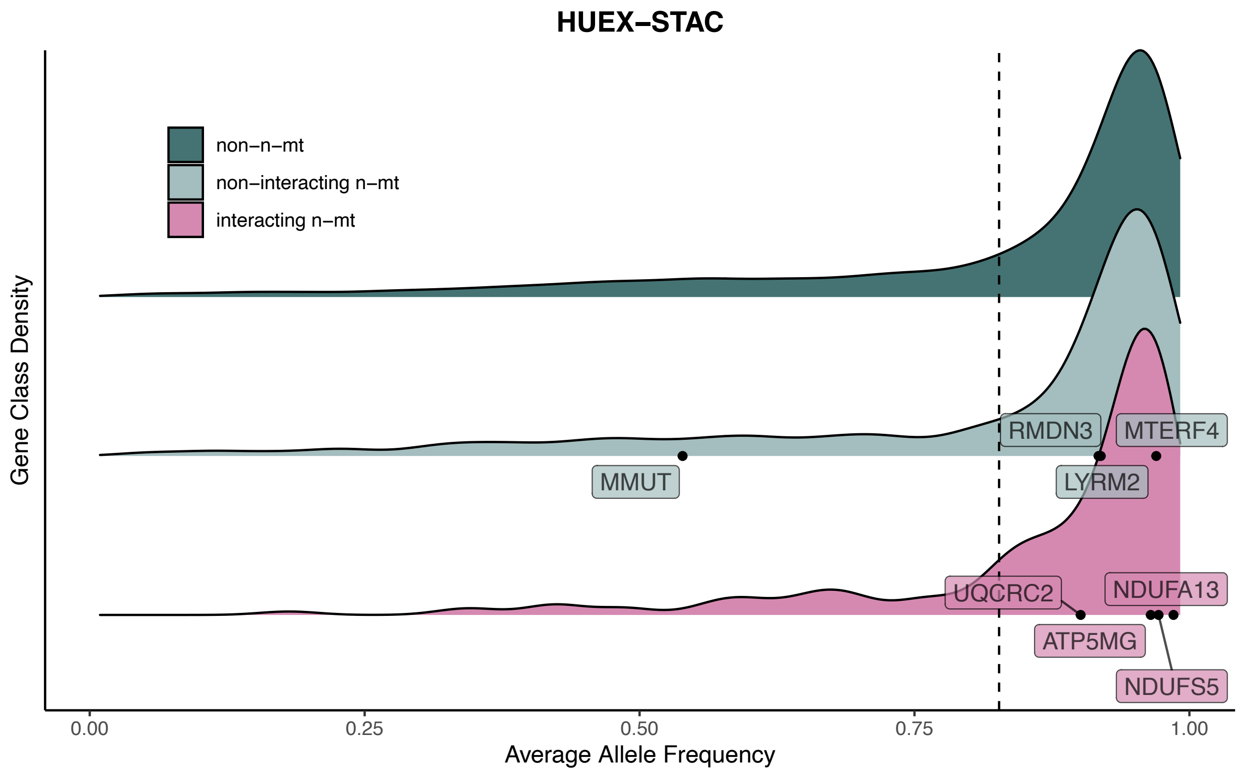

Supplemental Figure 5. Location of previously identified incompatibility genes [44] in the HUEX-STAC population X. cortezi *allele frequency values. Dashed line indicates genome-wide mean allele frequency.*

bioRxiv preprint doi: https://doi.org/10.1101/2025.06.30.662443; this version posted July 4, 2025. The copyright holder for this preprint (which was not certified by peer review) is the author/funder, who has granted bioRxiv a license to display the preprint in perpetuity. It is made available under aCC-BY 4.0 International license.

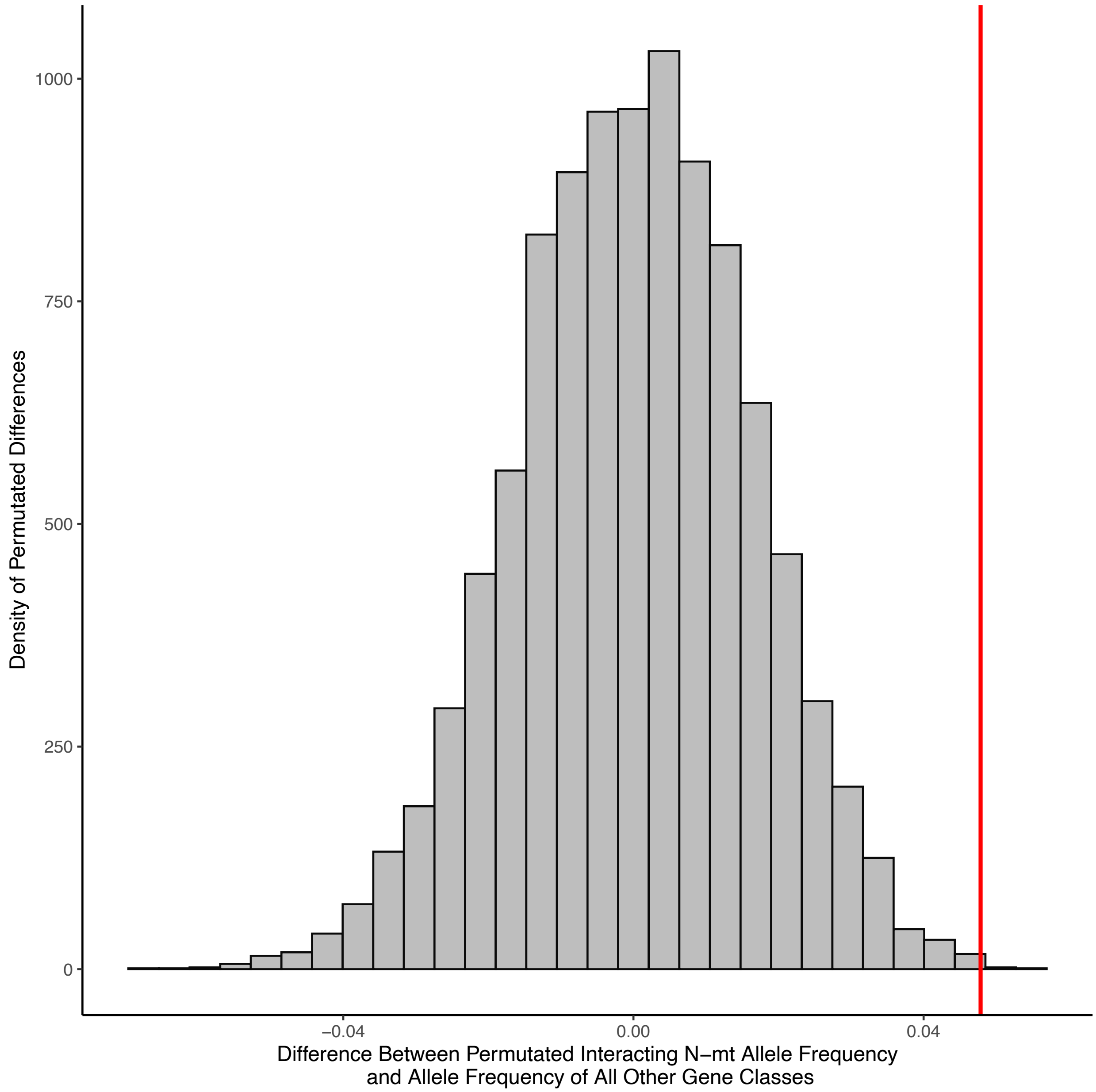

Supplemental Figure 6. Histogram demonstrating permuted differences in mean allele frequency between all interacting n-mts and the mean of all other genes. Red line represents the observed difference in interacting n-mt gene class and all other classes. In this permutation test, we found the interacting n-mt gene class to be different from the other two gene classes ($p = 5 \times 10^{-4}$), supporting the parametric analysis (ANOVA) in this population.

bioRxiv preprint doi: https://doi.org/10.1101/2025.06.30.662443; this version posted July 4, 2025. The copyright holder for this preprint (which was not certified by peer review) is the author/funder, who has granted bioRxiv a license to display the preprint in perpetuity. It is made available under aCC-BY 4.0 International license.

SUPPLEMENTAL TABLES

Supplemental Table 1. List of human interacting n-mt genes used in this study, modified from [49].

Uniprot ID	Entrez symbol	Gene symbol	Gene Name
P82663	64432	MRPS25	mitochondrial ribosomal protein S25
O15239	4694	NDUFA1	NADH:ubiquinone oxidoreductase subunit A1
Q9Y3B7	65003	MRPL11	mitochondrial ribosomal protein L11
O43678	4695	NDUFA2	NADH:ubiquinone oxidoreductase subunit A2
Q96EL2	64951	MRPS24	mitochondrial ribosomal protein S24
O95167	4696	NDUFA3	NADH:ubiquinone oxidoreductase subunit A3
Q9BYD2	65005	MRPL9	mitochondrial ribosomal protein L9
Q9BYD6	65008	MRPL1	mitochondrial ribosomal protein L1
Q9Y6G3	28977	MRPL42	mitochondrial ribosomal protein L42
P14854	1340	COX6B1	cytochrome c oxidase subunit 6B1
Q9Y2R9	51081	MRPS7	mitochondrial ribosomal protein S7
Q9NVS2	55168	MRPS18A	mitochondrial ribosomal protein S18A
P12074	1337	COX6A1	cytochrome c oxidase subunit 6A1
O00217	4728	NDUFS8	NADH:ubiquinone oxidoreductase core subunit S8
Q9BYN8	64949	MRPS26	mitochondrial ribosomal protein S26
P19404	4729	NDUFV2	NADH:ubiquinone oxidoreductase core subunit V2
Q86Y39	126328	NDUFA11	NADH:ubiquinone oxidoreductase subunit A11
Q9Y676	28973	MRPS18B	mitochondrial ribosomal protein S18B
O14949	27089	UQCRQ	ubiquinol-cytochrome c reductase complex III subunit VII
P30049	513	ATP5F1D	ATP synthase F1 subunit delta
P56381	514	ATP5F1E	ATP synthase F1 subunit epsilon
Q9H9J2	65080	MRPL44	mitochondrial ribosomal protein L44
P24539	515	ATP5PB	ATP synthase peripheral stalk-membrane subunit b
O75306	4720	NDUFS2	NADH:ubiquinone oxidoreductase core subunit S2
Q9UI09	55967	NDUFA12	NADH:ubiquinone oxidoreductase subunit A12
Q96GC5	51642	MRPL48	mitochondrial ribosomal protein L48
P05496	516	ATP5MC1	ATP synthase membrane subunit c locus 1
Q06055	517	ATP5MC2	ATP synthase membrane subunit c locus 2
O75489	4722	NDUFS3	NADH:ubiquinone oxidoreductase core subunit S3
P09001	11222	MRPL3	mitochondrial ribosomal protein L3
P48201	518	ATP5MC3	ATP synthase membrane subunit c locus 3
P49821	4723	NDUFV1	NADH:ubiquinone oxidoreductase core subunit V1
Q5JTZ9	57505	AARS2	alanyl-tRNA synthetase 2, mitochondrial
O43181	4724	NDUFS4	NADH:ubiquinone oxidoreductase subunit S4
O43920	4725	NDUFS5	NADH:ubiquinone oxidoreductase subunit S5
Q9Y3D9	51649	MRPS23	mitochondrial ribosomal protein S23
Q02221	1339	COX6A2	cytochrome c oxidase subunit 6A2
O75380	4726	NDUFS6	NADH:ubiquinone oxidoreductase subunit S6
P82914	64960	MRPS15	mitochondrial ribosomal protein S15
Q9NRX2	63875	MRPL17	mitochondrial ribosomal protein L17
P82912	64963	MRPS11	mitochondrial ribosomal protein S11
P15954	1350	COX7C	cytochrome c oxidase subunit 7C
P82933	64965	MRPS9	mitochondrial ribosomal protein S9
P82932	64968	MRPS6	mitochondrial ribosomal protein S6
Q9BYC9	55052	MRPL20	mitochondrial ribosomal protein L20
P82664	55173	MRPS10	mitochondrial ribosomal protein S10
P82675	64969	MRPS5	mitochondrial ribosomal protein S5
P24311	1349	COX7B	cytochrome c oxidase subunit 7B
O95363	10667	FARS2	phenylalanyl-tRNA synthetase 2, mitochondrial
P14406	1347	COX7A2	cytochrome c oxidase subunit 7A2
Q9Y2R5	51373	MRPS17	mitochondrial ribosomal protein S17
P24310	1346	COX7A1	cytochrome c oxidase subunit 7A1
P56385	521	ATP5ME	ATP synthase membrane subunit e

bioRxiv preprint doi: https://doi.org/10.1101/2025.06.30.662443; this version posted July 4, 2025. The copyright holder for this preprint (which was not certified by peer review) is the author/funder, who has granted bioRxiv a license to display the preprint in perpetuity. It is made available under aCC-BY 4.0 International license.

P09669	1345	COX6C	cytochrome c oxidase subunit 6C
Q9Y291	51650	MRPS33	mitochondrial ribosomal protein S33
P18859	522	ATP5PF	ATP synthase peripheral stalk subunit F6
Q9BZE1	51253	MRPL37	mitochondrial ribosomal protein L37
P82673	60488	MRPS35	mitochondrial ribosomal protein S35
Q9NSE4	55699	IARS2	isoleucyl-tRNA synthetase 2, mitochondrial
Q9UDW1	29796	UQCR10	ubiquinol-cytochrome c reductase, complex III subunit X
Q4U2R6	51258	MRPL51	mitochondrial ribosomal protein L51
O00483	4697	NDUFA4	NDUFA4 mitochondrial complex associated
Q9UHN1	11232	POLG2	DNA polymerase gamma 2, accessory subunit
Q16718	4698	NDUFA5	NADH:ubiquinone oxidoreductase subunit A5
P56181	4731	NDUFV3	NADH:ubiquinone oxidoreductase subunit V3
Q9BRJ2	84311	MRPL45	mitochondrial ribosomal protein L45
Q7Z2W9	219927	MRPL21	mitochondrial ribosomal protein L21
P54098	5428	POLG	DNA polymerase gamma, catalytic subunit
P82650	56945	MRPS22	mitochondrial ribosomal protein S22
O00411	5442	POLRMT	RNA polymerase mitochondrial
Q8IXM3	64975	MRPL41	mitochondrial ribosomal protein L41
Q9NQ50	64976	MRPL40	mitochondrial ribosomal protein L40
Q9P0J6	64979	MRPL36	mitochondrial ribosomal protein L36
Q96DV4	64978	MRPL38	mitochondrial ribosomal protein L38
Q9Y2Q9	28957	MRPS28	mitochondrial ribosomal protein S28
Q99766	27109	DMAC2L	distal membrane arm assembly component 2 like
Q9HD33	57129	MRPL47	mitochondrial ribosomal protein L47
Q8TCC3	51263	MRPL30	mitochondrial ribosomal protein L30
Q9Y3D3	51021	MRPS16	mitochondrial ribosomal protein S16
Q9P015	29088	MRPL15	mitochondrial ribosomal protein L15
P25705	498	ATP5F1A	ATP synthase F1 subunit alpha
Q8N5N7	54534	MRPL50	mitochondrial ribosomal protein L50
Q9Y3D5	51023	MRPS18C	mitochondrial ribosomal protein S18C
Q9P0M9	51264	MRPL27	mitochondrial ribosomal protein L27
Q9UGM6	10352	WARS2	tryptophanyl tRNA synthetase 2, mitochondrial
Q9BQC6	78988	MRPL57	mitochondrial ribosomal protein L57
P48047	539	ATP5PO	ATP synthase peripheral stalk subunit OSCP
O75251	374291	NDUFS7	NADH:ubiquinone oxidoreductase core subunit S7
Q9NP81	54938	SARS2	seryl-tRNA synthetase 2, mitochondrial
O75947	10476	ATP5PD	ATP synthase peripheral stalk subunit d
Q9NX14	54539	NDUFB11	NADH:ubiquinone oxidoreductase subunit B11
O75964	10632	ATP5MG	ATP synthase membrane subunit g
P49590	23438	HARS2	histidyl-tRNA synthetase 2, mitochondrial
P20674	9377	COX5A	cytochrome c oxidase subunit 5A
Q9BQ48	64981	MRPL34	mitochondrial ribosomal protein L34
Q92665	10240	MRPS31	mitochondrial ribosomal protein S31
Q9BYC8	64983	MRPL32	mitochondrial ribosomal protein L32
Q7L3T8	25973	PARS2	prolyl-tRNA synthetase 2, mitochondrial
O60783	63931	MRPS14	mitochondrial ribosomal protein S14
Q6YFQ2	125965	COX6B2	cytochrome c oxidase subunit 6B2
Q7Z7H8	124995	MRPL10	mitochondrial ribosomal protein L10
Q9NYK5	54148	MRPL39	mitochondrial ribosomal protein L39
Q15031	23395	LARS2	leucyl-tRNA synthetase 2, mitochondrial
Q7Z4L0	341947	COX8C	cytochrome c oxidase subunit 8C
Q9NWU5	29093	MRPL22	mitochondrial ribosomal protein L22
P49406	9801	MRPL19	mitochondrial ribosomal protein L19
Q9NP92	10884	MRPS30	mitochondrial ribosomal protein S30
P41250	2617	GARS1	glycyl-tRNA synthetase 1
Q9NX20	54948	MRPL16	mitochondrial ribosomal protein L16
Q9NZE8	51318	MRPL35	mitochondrial ribosomal protein L35
Q16540	6150	MRPL23	mitochondrial ribosomal protein L23
Q9Y2S7	26073	POLDIP2	DNA polymerase delta interacting protein 2

bioRxiv preprint doi: https://doi.org/10.1101/2025.06.30.662443; this version posted July 4, 2025. The copyright holder for this preprint (which was not certified by peer review) is the author/funder, who has granted bioRxiv a license to display the preprint in perpetuity. It is made available under aCC-BY 4.0 International license.

O14957	10975	UQCR11	ubiquinol-cytochrome c reductase, complex III subunit XI
Q96I59	79731	NARS2	asparaginyl-tRNA synthetase 2, mitochondrial
Q96GW9	92935	MARS2	methionyl-tRNA synthetase 2, mitochondrial
Q13084	10573	MRPL28	mitochondrial ribosomal protein L28
Q7Z7F7	128308	MRPL55	mitochondrial ribosomal protein L55
P08574	1537	CYC1	cytochrome c1
O75394	9553	MRPL33	mitochondrial ribosomal protein L33
P56134	9551	ATP5MF	ATP synthase membrane subunit f
Q5JPH6	124454	EARS2	glutamyl-tRNA synthetase 2, mitochondrial
Q5T160	57038	RARS2	arginyl-tRNA synthetase 2, mitochondrial
Q9H0U6	29074	MRPL18	mitochondrial ribosomal protein L18
P22695	7385	UQCRC2	ubiquinol-cytochrome c reductase core protein 2
P31930	7384	UQCRC1	ubiquinol-cytochrome c reductase core protein 1
P47985	7386	UQCRFS1	ubiquinol-cytochrome c reductase, Rieske iron-sulfur polypeptide 1
P07919	7388	UQCRH	ubiquinol-cytochrome c reductase hinge protein
Q9BYD1	28998	MRPL13	mitochondrial ribosomal protein L13
Q96A35	79590	MRPL24	mitochondrial ribosomal protein L24
Q92552	23107	MRPS27	mitochondrial ribosomal protein S27
O95299	4705	NDUFA10	NADH:ubiquinone oxidoreductase subunit A10
Q6P1L8	64928	MRPL14	mitochondrial ribosomal protein L14
Q9BW92	80222	TARS2	threonyl-tRNA synthetase 2, mitochondrial
O14561	4706	NDUFAB1	NADH:ubiquinone oxidoreductase subunit AB1
Q96KJ9	84701	COX4I2	cytochrome c oxidase subunit 4I2
O95178	4708	NDUFB2	NADH:ubiquinone oxidoreductase subunit B2
O43676	4709	NDUFB3	NADH:ubiquinone oxidoreductase subunit B3
Q9Y2Z4	51067	YARS2	tyrosyl-tRNA synthetase 2
Q5T653	51069	MRPL2	mitochondrial ribosomal protein L2
Q8N983	84545	MRPL43	mitochondrial ribosomal protein L43
Q9Y375	51103	NDUFAF1	NADH:ubiquinone oxidoreductase complex assembly factor 1
P56556	4700	NDUFA6	NADH:ubiquinone oxidoreductase subunit A6
O95182	4701	NDUFA7	NADH:ubiquinone oxidoreductase subunit A7
P51970	4702	NDUFA8	NADH:ubiquinone oxidoreductase subunit A8
Q15046	3735	KARS1	lysyl-tRNA synthetase 1
Q86TS9	122704	MRPL52	mitochondrial ribosomal protein L52
Q16795	4704	NDUFA9	NADH:ubiquinone oxidoreductase subunit A9
P82930	65993	MRPS34	mitochondrial ribosomal protein S34
Q5ST30	57176	VARS2	valyl-tRNA synthetase 2, mitochondrial
Q9H2W6	26589	MRPL46	mitochondrial ribosomal protein L46
P52815	6182	MRPL12	mitochondrial ribosomal protein L12
P82921	54460	MRPS21	mitochondrial ribosomal protein S21
O15235	6183	MRPS12	mitochondrial ribosomal protein S12
P13073	1327	COX4I1	cytochrome c oxidase subunit 4I1
O96000	4716	NDUFB10	NADH:ubiquinone oxidoreductase subunit B10
Q6PI48	55157	DARS2	aspartyl-tRNA synthetase 2, mitochondrial
O43677	4717	NDUFC1	NADH:ubiquinone oxidoreductase subunit C1
Q9BYD3	51073	MRPL4	mitochondrial ribosomal protein L4
Q96EL3	116540	MRPL53	mitochondrial ribosomal protein L53
O95298	4718	NDUFC2	NADH:ubiquinone oxidoreductase subunit C2
P28331	4719	NDUFS1	NADH:ubiquinone oxidoreductase core subunit S1
Q13405	740	MRPL49	mitochondrial ribosomal protein L49
Q6P161	116541	MRPL54	mitochondrial ribosomal protein L54
Q9P0J0	51079	NDUFA13	NADH:ubiquinone oxidoreductase subunit A13
O95168	4710	NDUFB4	NADH:ubiquinone oxidoreductase subunit B4
O43674	4711	NDUFB5	NADH:ubiquinone oxidoreductase subunit B5
Q9Y399	51116	MRPS2	mitochondrial ribosomal protein S2
P06576	506	ATP5F1B	ATP synthase F1 subunit beta
O95139	4712	NDUFB6	NADH:ubiquinone oxidoreductase subunit B6
P17568	4713	NDUFB7	NADH:ubiquinone oxidoreductase subunit B7
P10606	1329	COX5B	cytochrome c oxidase subunit 5B

bioRxiv preprint doi: https://doi.org/10.1101/2025.06.30.662443; this version posted July 4, 2025. The copyright holder for this preprint (which was not certified by peer review) is the author/funder, who has granted bioRxiv a license to display the preprint in perpetuity. It is made available under aCC-BY 4.0 International license.

O95169	4714	NDUFB8	NADH:ubiquinone oxidoreductase subunit B8
P36542	509	ATP5F1C	ATP synthase F1 subunit gamma
Q9Y6M9	4715	NDUFB9	NADH:ubiquinone oxidoreductase subunit B9

bioRxiv preprint doi: https://doi.org/10.1101/2025.06.30.662443; this version posted July 4, 2025. The copyright holder for this preprint (which was not certified by peer review) is the author/funder, who has granted bioRxiv a license to display the preprint in perpetuity. It is made available under aCC-BY 4.0 International license.

Supplemental Table 2. Summary statistics of mitonuclear association values for each hybrid population.

Population	Statistic	Gene Class	Count	Mean	Standard Deviation
**CALL**	D'	Interacting n-mt	148	0.370	0.086
		Non-interacting n-mt	883	0.369	0.094
		Non-n-mt	19,312	0.368	0.097
	r^2	Interacting n-mt	148	0.084	0.058
		Non-interacting n-mt	883	0.086	0.054
		Non-n-mt	19,312	0.088	0.058
**CHAF**	D'	Interacting n-mt	148	0.397	0.169
		Non-interacting n-mt	883	0.396	0.174
		Non-n-mt	19,307	0.396	0.177
	r^2	Interacting n-mt	148	0.030	0.024
		Non-interacting n-mt	883	0.028	0.022
		Non-n-mt	19.307	0.028	0.023
**HUEX-STAC**	Allele Frequency	Interacting n-mt	162	0.874	0.150
		Non-interacting n-mt	947	0.818	0.217
		Non-n-mt	20544	0.827	0.206

bioRxiv preprint doi: https://doi.org/10.1101/2025.06.30.662443; this version posted July 4, 2025. The copyright holder for this preprint (which was not certified by peer review) is the author/funder, who has granted bioRxiv a license to display the preprint in perpetuity. It is made available under aCC-BY 4.0 International license.

Supplemental Table 3. Measures of mitonuclear association for incompatible n-mt genes previously identified by Robles et al. [44].

		**CALL**[c]		**CHAF**[c]		**HUEX-STAC**[c]
**Gene**[a]	**Incompatible mtDNA**[b]	***D′***	***r^2***	***D′***	***r^2***	**Allele frequency**
NDUFA13 (g3279.t1)	*X. malinche*	0.57 (2.08)	0.18 (1.65)	0.54 (0.83)	0.15 (5.37)	0.99 (0.77)
MTERF4 (g3322.t1)	*X. malinche*	0.58 (2.16)	0.14 (0.82)	0.59 (1.12)	0.19 (6.86)	0.97 (0.69)
ATP5MG (g6088.t1)	*X. malinche*	ND	ND	ND	ND	0.96 (0.67)
NDUFS5 (g8054.t1)	bidirectional	0.65 (2.91)	0.40 (5.34)	0.73 (1.91)	0.16 (5.58)	0.97 (0.70)
MMUT (g15308.t1)	bidirectional	0.42 (0.57)	0.04 (-0.87)	0.00 (-2.21)	0.00 (-1.22)	0.54 (-1.40)
SMIM8 (g15060.t1)	*X. birchmanni*	0.59 (2.26)	0.27 (3.10)	0.67 (1.53)	0.05 (0.89)	ND
LYRM2 (g15051.t1)	*X. birchmanni*	0.49 (1.29)	0.22 (2.30)	0.59 (1.07)	0.04 (0.59)	0.92 (0.45)
RMDN3 (g15056.t1)	*X. birchmanni*	0.58 (2.22)	0.27 (3.15)	0.58 (1.04)	0.04 (0.44)	0.92 (0.44)
UQCRC2 (g11575.t1)	*X. birchmanni*	0.33 (-0.40)	0.04 (-0.76)	0.11 (-1.60)	0.00 (-1.14)	0.90 (0.36)

[a]Human gene names are given with the X. birchmanni annotation ID indicated in parentheses. UQCR11 has also been identified as a gene involved in mitonuclear incompatibilities [41], but it was not included in the annotation used in this analysis.
[b]Species listed indicate the mtDNA that is incompatible with the other species' n-mt allele. For example, X. malinche means the X. malinche mtDNA is incompatible with X. birchmanni ancestry at NDUFA13. Bidirectional means the incompatibility occurs for both mtDNAs..
[c]Values are reported for each metric of mitonuclear association (*D′*, r^2, and allele frequency), and the corresponding Z-score for each value is indicated in parentheses, which was calculated by subtracting the mean value and dividing by the standard deviation.

bioRxiv preprint doi: https://doi.org/10.1101/2025.06.30.662443; this version posted July 4, 2025. The copyright holder for this preprint (which was not certified by peer review) is the author/funder, who has granted bioRxiv a license to display the preprint in perpetuity. It is made available under aCC-BY 4.0 International license.

Supplemental Table 4. Results of Fisher's exact test for enrichment of interacting n-mt genes in top 1% of genome-wide association statistics.

Population	Test	Result
CALL	One-sided Fisher's Exact Test for Count Data on D' values	$p = 0.42$
	One-sided Fisher's Exact Test for Count Data r^2 values	$p = 0.22$
CHAF	One-sided Fisher's Exact Test for Count Data on D' values	$p = 0.66$

bioRxiv preprint doi: https://doi.org/10.1101/2025.06.30.662443; this version posted July 4, 2025. The copyright holder for this preprint (which was not certified by peer review) is the author/funder, who has granted bioRxiv a license to display the preprint in perpetuity. It is made available under aCC-BY 4.0 International license.

Supplemental Table 5. Studies investigating differential effects of n-mt and non-n-mt gene groups or genomic windows.

Citation	Admixture category[a]	Hybridizing groups	Segregating mtDNA?[b]	Disproportionate n-mt gene effect?[c]
**Mollusks**				
Fields et al. 2024 [59]	Historical introgression	*Potamopyrgus estuarinus* and *P. kaitunuparaoa*	No	Yes
**Fish**				
Kato et al. 2023 [60]	Historical introgression	*Chaenogobius annularis* and a ghost lineage	No	No
**Mammals**				
Kwon et al. 2022 [61]	Interspecific hybridization	*Bos taurus* and *B. indicus*	No	Yes
Shi et al. 2025 [62]	Interspecific hybridization	*B. taurus* and *B. indicus*	No	No[d]
Jensen et al. 2023 [63]	Historical introgression	*Allochrocebus* and *Chlorocebus* lineages	No	Yes
Zhu & Evans 2023 [64]	Historical introgression	*Macaca arctoides* and *M. fascicularis aurea*	No	No
Evans et al. 2021 [65]	Interspecies individual hybrid	*M. tonkeana* and *M. maura*	This individual has *M. tonkeana* mtDNA	N/A[f]
	Interspecies individual hybrid	*M. tonkeana* and *M. hecki*	This individual has *M. hecki* mtDNA	N/A[f]
Bailey & Stevison 2021 [66]	Interspecific hybridization	*M. arctoides* and *M. sinica* or *M. fascicularis*	No	No
Sloan et al. 2015 [49]	Intraspecific admixture between populations	*Homo sapiens*	Yes	No
Sharbrough et al. 2017 [67]	Historical introgression	*H. sapiens* and *H. neanderthalensis*	No	Yes
	Historical introgression	*H. sapiens* and Denisovans	No	No

bioRxiv preprint doi: https://doi.org/10.1101/2025.06.30.662443; this version posted July 4, 2025. The copyright holder for this preprint (which was not certified by peer review) is the author/funder, who has granted bioRxiv a license to display the preprint in perpetuity. It is made available under aCC-BY 4.0 International license.

Zaidi & Makova 2019 [68]	Intraspecific admixture between populations	*H. sapiens* African American and European	Yes	Yes
	Intraspecific admixture between populations	*H. sapiens* Puerto Rican and European	Yes	Yes
	Intraspecific admixture between populations	*H. sapiens* Caribbean and European	Yes	No
	Intraspecific admixture between populations	*H. sapiens* Columbian and European	Yes	No
	Intraspecific admixture between populations	*H. sapiens* Mexican and European	Yes	No
	Intraspecific admixture between populations	*H. sapiens* Peruvian and European	Yes	No
**Birds**				
Lopez et al. 2021 [55]	Intraspecific admixture between populations	*Poephila acuticauda acuticauda* and *P. a. hecki*	Yes	Yes[e]
Morales et al. 2018 [69]	Intraspecific admixture between populations	northern and southern populations of *Eopsaltria australis*	Yes	Yes, but see [56]
Low et al. 2024 [56]	Intraspecific admixture between populations	inland and coastal mitochondrial lineages of *E. australis*	Yes	Yes[e]
Rancilhac et al. 2024 [70]	Interspecific hybridization	*Pogoniulus pusillus* and *P. chrysoconus*	Yes	No
Musher et al. 2024 [71]	Intraspecific admixture	*Thamnophilus aethiops*	Yes	No

These papers were collected, using two groups of search terms to identify published articles that included similar comparisons between n-mt and non-n-mt genes in the context of hybridization. The first search terms were "n-mt" AND ("reproductive isolation" OR "speciation") AND “hybrid” on Google Scholar in October 2024. Inclusion criteria for the resulting hits were that the work (1) categorized genes into n-mt and non-n-mt classes, (2) measured association between n-mt genetic variants and mitochondrial haplotypes, and (3) was focused on current or ancient admixture of genomes (via hybridization or introgression). Papers were excluded if the only aim was to use phylogenetic analyses to study mutation rates or phylogenetic discordance between mtDNA and non-n-mt loci. This search (conducted on 18 October 2024) identified 193 possible articles, but only 10 met our inclusion criteria. We then performed a separate search using the terms “mito*nuclear” AND (“reproductive isolation” OR “speciation.”), returning 57 articles, but none that met our inclusion criteria. We supplemented this list with six additional papers that were not returned as hits in the above searches but that we encountered as citations in other studies or during unrelated literature searches. The combination of search terms and narrow inclusion criteria likely excluded some relevant studies from this table, but the goal was not to capture all studies pertinent to genomic analysis of mitonuclear interactions.

[a]Hybridization systems were categorized as being either intraspecific admixture between populations, interspecific hybridization, or historical introgression to indicate timing of hybridization and how removed from initial admixture a population may be.

bioRxiv preprint doi: https://doi.org/10.1101/2025.06.30.662443; this version posted July 4, 2025. The copyright holder for this preprint (which was not certified by peer review) is the author/funder, who has granted bioRxiv a license to display the preprint in perpetuity. It is made available under aCC-BY 4.0 International license.

[b]Indication of whether the mtDNA from the hybrid source populations or species are still segregating in the hybrid system investigated.
[c]Investigations that found n-mt gene groups or genomic windows to share ancestry more than non-n-mt groups or windows were given a ‘yes’ to indicate whole-genome based methods successfully identified effects of n-mt selection.
[d]This study instead found evidence of enrichment for mismatch of n-mt and mtDNA ancestry.
[e]These studies find positive relationships between n-mt and mtDNA ancestry, but these relationships also include matching of sex chromosome ancestry or that n-mt genes are located in regions of a chromosome associated with sex determination.
[f]Because these studies only determined n-mt disproportionality with a single individual, they are omitted from our investigation into whether a fixing a mitochondrial haplotype affects the sensitivity of genome-wide tests of shared n-mt and mtDNA ancestry.
